# Effects of Velocity-Based versus Percentage-Based Resistance Training on Explosive Neuromuscular Adaptations and Anaerobic Power in Sport-College Female Basketball Players

**DOI:** 10.3390/healthcare11040623

**Published:** 2023-02-20

**Authors:** Mingyang Zhang, Duanying Li, Jiaxin He, Xingyue Liang, Dongyu Li, Wenfeng Song, Shicong Ding, Jie Shu, Xiaoning Sun, Jian Sun

**Affiliations:** 1 Digital Physical Training Laboratory, Guangzhou Sport University, Guangzhou 510500, China; 2 Department of Physical Education, Guangzhou Sport University, Guangzhou 510500, China; 3 School of Athletic Training, Guangzhou Sport University, Guangzhou 510500, China

**Keywords:** load-velocity relationship, autoregulation, load monitoring, fixed-loading, resistance training, Wingate anaerobic performance

## Abstract

The purpose of this study was to compare the impact of velocity-based resistance training (VBRT) and percentage-based resistance training (PBRT) on anaerobic ability, sprint performance, and jumping ability. Eighteen female basketball players from a Sport College were randomly divided into two groups: VBRT (n = 10) and PBRT (n = 8). The six-week intervention consisted of two sessions per week of free-weight back squats with linear periodization from 65% to 95%1RM. In PBRT, the weights lifted were fixed based on 1RM percentage, while in VBRT, the weights were adjusted based on individualized velocity profiles. The T-30m sprint time, relative power of countermovement jump (RP-CMJ), and Wingate test were evaluated. The Wingate test assessed peak power (PP), mean power (MP), fatigue index (FI), maximal velocity (Vmax), and total work (TW). Results showed that VBRT produced a very likely improvement in RP-CMJ, Vmax, PP, and FI (Hedges’ g = 0.55, 0.93, 0.68, 0.53, respectively, *p* < 0.01). On the other hand, PBRT produced a very likely improvement in MP (Hedges’ g = 0.38) and TW (Hedges’ g = 0.45). Although VBRT showed likely favorable effects in RP-CMJ, PP, and Vmax compared to PBRT (*p* < 0.05 for interaction effect), PBRT produced greater improvements in MP and TW (*p* < 0.05 for interaction effect). In conclusion, PBRT may be more effective in maintaining high-power velocity endurance, while VBRT has a greater impact on explosive power adaptations.

## 1. Introduction

“Traditionally, resistance training (RT) has been prescribed based on a percentage of an individual’s one-repetition maximum (1RM), referred to as percentage-based resistance training (PBRT) [1]. This involves setting a fixed load based on a baseline %1RM before training, meaning the intensity is determined by the individual’s %1RM. However, PBRT is unable to account for changes in muscle performance caused by life stressors and fatigue [2]. With the rise of linear position transducers (LPTs), strength training coaches can now gather real-time and kinetic data, leading to the widespread use of velocity-based resistance training (VBRT) [2,3]. VBRT is a novel form of autoregulated RT that adjusts intensity and volume based on an individualized load-velocity profile (LVP) regression equation [2,4,5]. This involves using the mean concentric velocity (MCV) of the first repetition as a measure of performance to adjust the training load. Trainers perform the exercise with maximal effort and the velocity of the concentric phase is recorded across different loads. Thresholds are established at each relative load or velocity loss, which are then used to adjust the subsequent training load and control the training volume [6]. By monitoring MCV data based on LVP, VBRT allows for training loads to be adjusted based on the athlete’s physiological state and strength performance [7]. Notably, in contrast to PBRT, VBRT offers a more personalized approach to load prescription [2].

Recently, there have been several controlled trials that have compared the effects of VBRT and PBRT on physical performance measures such as strength, linear sprint, change of direction, jump, and aerobic endurance [8,9,10]. This comparison has become a focus in the field of strength and conditioning, and while these studies provide useful insights for coaches, the results in terms of muscle strength remain controversial and the mechanisms behind the adaptations are not fully understood [1,3,9,11]. To further shed light on this topic, this study aims to compare the two training protocols on lower limb muscular function and power performance in greater depth. The originality of this study lies in its exploration of the effects of VBRT and PBRT on anaerobic performance adaptations, an area that has not been studied in depth before.

Basketball is a sport that requires high levels of intensity and is largely based on anaerobic metabolism. Anaerobic power and capacity play a crucial role in determining the physical fitness and overall game performance of basketball players, particularly in defensive and offensive transitions [12]. To assess anaerobic performance in professional basketball players, the Wingate anaerobic power test (WAnT) has been widely used due to its reliability. The results of a study by Apostolidis et al. [13] showed a strong correlation between the mean power in the WAnT and the performance of basketball players, including control dribbling and high-intensity shuttle running.

This study aimed to compare the effects of VBRT and PBRT on lower-limb power and anaerobic performance in off-season female basketball players from a Sport College over a six-week linear mesocycle. The hypothesis was that VBRT, based on recent studies [1,9,10], would result in greater improvements in anaerobic performance compared to PBRT.

## 2. Materials and Methods

### 2.1. Participants

The study recruited 18 female basketball players from the Sport College Basketball Association (SCBA) Championship winning team. The players, who had an average age of 22.3 ± 1.8 years, height of 169.7 ± 7 cm, and body mass of 60.4 ± 5.8 kg, were randomly assigned to either the VBRT group (n = 10) or the PBRT group (n = 8) using card markers drawn by an uninformed researcher. The distribution of playing positions was equal among the groups to reduce the impact of position on anaerobic capacity [14]. The inclusion criteria were age over 18 years, at least 2 years of RT experience, no musculoskeletal injuries in the past 6 months, completion of a 10-week basketball training prior to the study, and a negative result from the Functional Movement Screen (FMS) test.

Participants were required to provide written informed consent prior to participating in the study. The study was approved by the local Ethics Committee and was in accordance with the Declaration of Helsinki. This ensured that the study was conducted in a fair and ethical manner, with the participants’ wellbeing and rights being of utmost importance.

### 2.2. Experimental Design

**Figure 1:**
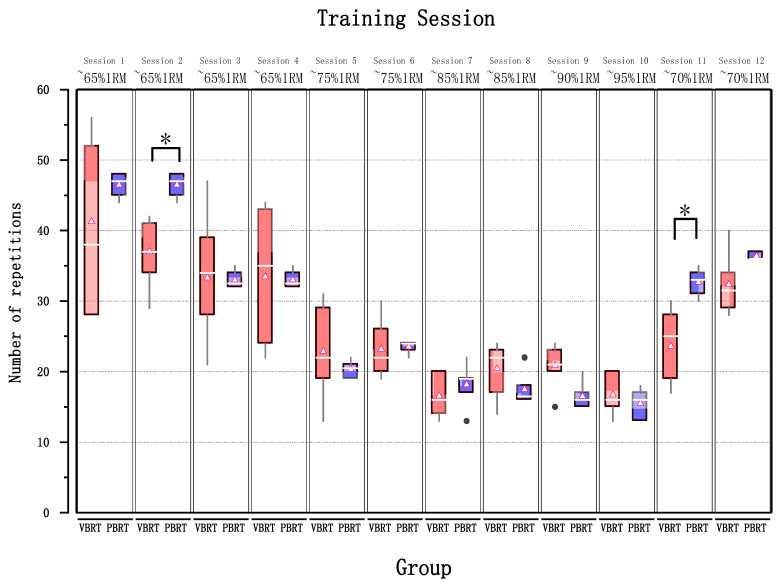
Number of repetitions in the squat exercise performed between VBRT and PBRT. Data are mean ± SD. VBRT: velocity-based resistance training. PBRT: percentage-based (%1RM) resistance training. Statistically significant differences between groups: * *p* < 0.05.

All participants took part in a 6-week training program, which was conducted twice a week on Monday and Wednesday and followed a progressive mesocycle design. The program consisted of three load phases, each with specific training objectives (as shown in Figure 1). Participants performed two RT programs that only varied in amount of weight lifted and number of repetitions (velocity-based vs. percentage-based). The training volume in both programs corresponded to the number of repetitions. Additionally, both groups were verbally encouraged to perform a maximal voluntary contraction at the concentric phase with a standardized body posture during RT. The RT sessions were completed in the afternoon (3:30–4:00 p.m.), were separated by 48 h, and avoided holidays. Both RT programs consisted of free-weight back squats and bench presses and were supervised and monitored by two conditioning training coaches.

All tests were conducted in a laboratory setting, with a minimum of 48 h of rest and under similar time (±0.5 h) and environmental conditions (~27 °C and ~68% humidity). The outcomes of anaerobic performance, sprint, jump performance, and body composition were evaluated at the baseline (before randomization) and at the end of the intervention. The training loads (weights and repetitions) and kinematic data were continuously recorded during each session.

### 2.3. Testing Procedure

Before the baseline assessment, participants were asked to come to the laboratory and complete an informed consent form and questionnaire. Their personal information such as age, height, and weight was recorded, and a standardized warm-up was conducted. The warm-up included fascial relaxation, self-prescribed dynamic stretching, activation exercises, and barbell mobility work, which lasted approximately 20 min. After the warm-up, participants underwent a familiarization session with all laboratory tests to ensure they fully understood the testing procedures and to guarantee that they adhered to the strict technical requirements during the tests. This was followed by a barbell back squat repetition session to familiarize the participants with the equipment and technique. 

All participants who met the inclusion criteria were permitted to participate in the study after the familiarization. Outcomes were evaluated at week 1 and week 7, where participants came to the laboratory to complete three sessions of assessments. These included body composition and 1 repetition maximum (1RM) assessment in session 1, jump, sprint test, and LVP assessment in session 2, and Wingate anaerobic test in session 3. Both baseline and post-test followed the same standardized warm-up protocol. The 1RM and LVP assessments were used to determine the training loads, but only performed at baseline. To minimize experimental error, the basketball coaches instructed all players to maintain a consistent diet and sleep pattern and monitored their additional resistance training during the intervention.

### 2.4. Measurements

#### 2.4.1. Anaerobic Power Performance

All participants performed the Wingate Anaerobic Test (WAnT) on a mechanically braked cycle ergometer (Monark© 894E, Varberg, Sweden). Participants were instructed to remain seated and complete a standardized warm-up, which involved riding at 60 revolutions per minute for 3–4 min at 60W, followed by two 5-s no-load sprints at 90 rpm. After a brief interval, participants began a 30-s all-out effort to maintain maximum speed with a resistance of 7.5% body mass. Verbal encouragement was provided throughout the test.

The peak power (PP), mean power (MP), and fatigue index (FI) were recorded and analyzed. The data was collected in the laboratory and processed using the Monark software.

#### 2.4.2. Jump and Sprint Testing

Relative peak power during a countermovement jump (RP-CMJ) and sprint ability were used as markers of explosive lower-limb power and athletic performance in basketball players. The RP-CMJ was conducted in three trials, with a 30-s rest in between, using the Smartjump wireless test mat (SmartJump; FusionSport, Coopers Plains, Queensland, Australia). Participants placed their hands on their hips, lowered themselves to the optimal level, and then jumped as high as possible. Following the CMJ, the participants completed three 30-m sprint trials with a 3-min rest in between. Sprint time was measured using the Smartspeed fusion sport (2 smart scans and pro, Australia), and a 1.5-m light gate was set up for the 30-m distance. The highest RP-CMJ and the fastest sprints were selected for further analysis. The test-retest reliability was high, with a coefficient variation of 0.99% and 1.4% for the T-30m and RP-CMJ, respectively. The intraclass correlation coefficients (ICC) with 95% confidence intervals (CI) were 0.968 (95% CI: 0.933–0.987) for T-30m, and 0.991 (95% CI: 0.981–0.996) for RP-CMJ.

#### 2.4.3. Body Composition

Body composition was evaluated using a bioelectrical impedance analysis (InBody 770 Body Composition Analyzer, Biospace, Seoul, South Korea) [15]. Relevant parameters, including muscle mass (MM), free-fat mass (FFM), and body mass index (BMI) were measured. Body composition assessments were performed at least 3 days after the last training session. The assessment was performed before warm-up and there were no cases of menstrual period. The laboratory had an internal temperature range of 24–26 °C. All participants were required to participate in body composition assessments in a fasted and dehydrated state (no prior water or food for 4 h).

#### 2.4.4. 1-RM Assessment

Body composition was evaluated using a bioelectrical impedance analysis device (InBody 770 Body Composition Analyzer, Biospace, Seoul, South Korea). The parameters measured included muscle mass (MM), free-fat mass (FFM), and body mass index (BMI). The assessments were performed at least 3 days after the participants’ last training sessions, before their warm-ups. To minimize any impact of menstrual cycle, all participants were assessed when not in their menstrual period. The laboratory was maintained at a temperature range of 24–26 °C, and participants were required to be in a fasted and dehydrated state (no food or water consumed for 4 h prior) during the assessments.

#### 2.4.5. LVP Assessment

The LPT was used to measure MCV during the free-weight back squat. Participants performed a protocol that included sets of 3 reps at 40%, 60%, 80%, and 90% of their baseline 1RM, with three minutes of rest between sets. The fastest MCV was used to calculate the LVP regression equation [9]. The individualized LVPs were determined by plotting MCV against load and applying a line of best fit, using the Gymaware cloud’s reporter. The MCV corresponding to 65–95% of 1RM was used to adjust daily lifted weights during the VBRT.

### 2.5. Resistance Training Program

The PBRT was based on a fixed load calculated from the baseline 1RM [11]. In contrast, the VBRT group performed auto-regulation training, adjusting the weight and reps according to the achieved Mean Concentric Velocity (MCV) and the target velocity from the Load-Velocity Profile (LVP). The VBRT group’s session target velocity was equivalent to the relative load in the PBRT group [9], albeit with different lifted weights. The specific velocity zones for each repetition and relative load were calculated for the VBRT group. If the MCV was 0.06 or 0.12 m/s above or below the target velocity, the next set weight was adjusted by 5% or 10% of the 1-RM [9]. The training information was provided to the strength coaches through visual and audio feedback.

During each training session, the average velocity was continuously monitored, and the appropriate load adjustments were made for each participant in the VBRT group. To ensure consistency, all participants utilized 20 kg barbells. The VBRT group also used LPTs (Gymaware Power Tool; Kinetic Performance Technologies, Australia), attached 60 cm to the right of the barbell center, to collect real-time concentric velocity data during the back squat exercise in every session.

### 2.6. Statistical Analysis

The descriptive statistics were presented as mean (standard deviation) or median (range). Test-retest reliability was evaluated through coefficient of variation (CV) and ICC with 95% CI. The one-way random effects model was used for this evaluation. To check for normality, the Shapiro-Wilk test was used, and Levene’s test was used to determine homogeneity of variance. In case of abnormal distribution or heterogeneity, the Mann-Whitney U test and Wilcoxon test were used to examine within-group and between-group differences, respectively. However, if the data was normally distributed and had homogeneous variance, the *T*-test was used. A two-way analysis of variance (ANOVA) with repeated measures was performed to analyze the time effects and time x group interactions to identify the best performing group. If there were significant group interactions, between-group comparisons were performed with the Bonferroni post-hoc test.

Statistical significance was determined at a two-tailed *p* < 0.05. In addition, the clinical significance of the effect size (ES) of the within-group and between-group differences was evaluated using magnitude-based inference [16]. The standard mean difference (SMD), calculated as the mean change divided by the baseline standard deviation, and its associated measures, including the smallest worthwhile change (0.2 times the baseline standard deviation) and Hedges’ g, were computed and used to determine the magnitude of the difference [16]. The effect size was calculated based on the Cohen scale, with positive SMD values indicating effects that favored the VBRT group, while negative SMD values indicated effects favoring the PBRT group. The magnitude of the effect was considered trivial (SMD < 0.2), small (0.2 to 0.59), moderate (0.6 to 1.19), or large (1.2 to 2.0) [16]. The likelihood of harmful or beneficial effects was estimated as almost certainly not likely (less than 0.5%), very unlikely (0.5% to 5%), unlikely (5% to 25%), possible (25% to 75%), likely (75% to 95%), very likely (95% to 99%), or almost certain (greater than 99.5%) [16]. In cases where both the harmful and beneficial changes were greater than 5%, the difference was considered unclear. The magnitude-based inferential statistics and intraclass correlation coefficients were calculated using a custom spreadsheet [17], with data analysis performed using jamovi 2.2.2 and R.

## 3. Results

No significant differences between VBRT and PBRT were found at baseline for all descriptive variables (Table 1).

### 3.1. Training Loads

The training data is depicted in Figure 1. After a six-week intervention, the VBRT group performed fewer repetitions compared to the PBRT group (31.1 ± 8.7 vs. 33.9 ± 12.17, respectively; *p* < 0.05). However, no significant difference was observed in the average weight lifted between the two groups (60.7 ± 11.9 vs. 63 ± 11.9, *p* > 0.05). The VBRT group experienced a higher intensity in weightlifting, as evidenced by a higher %1RM (80.2 ± 5.9% vs. 72 ± 6.5%, respectively) compared to the PBRT group.

### 3.2. Body Composition

No significant within-group differences were observed from baseline to post-intervention, and no time or group by time interaction effect was found for any of the lower limb muscle mass related parameters (Table 2).

### 3.3. WAnT Performance

**Figure 2:**
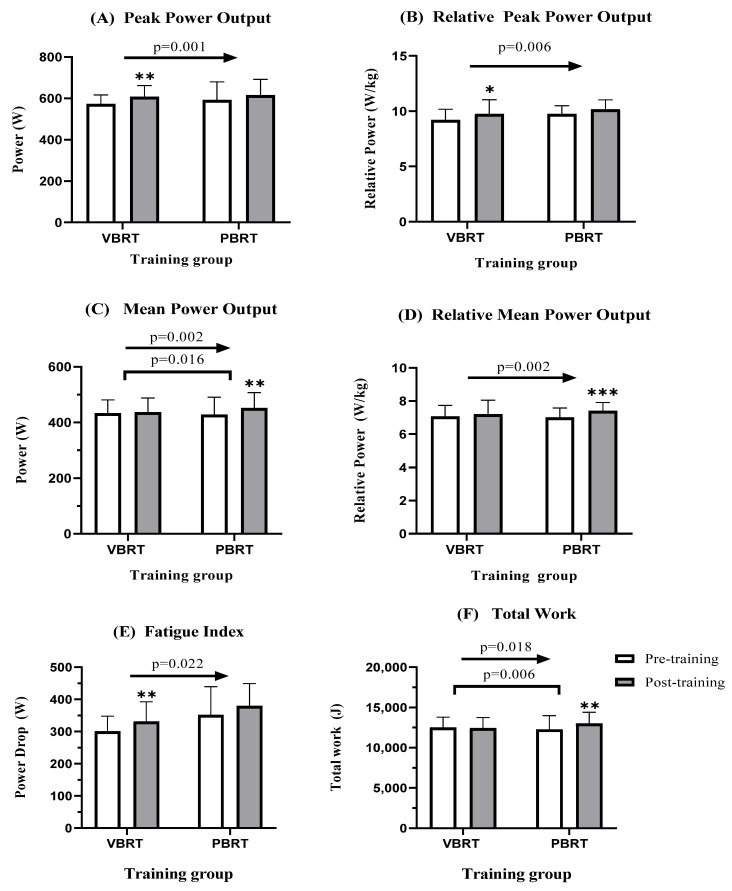
Mean changes in absolute (**A**) and relative peak power output (**B**), absolute (**C**) and relative mean power output (**D**), fatigue index (**E**) and total work (**F**) for the Wingate anaerobic power test after 6 weeks of training. * indicates significant difference baseline vs post-intervention: * *p* < 0.05, ** *p* < 0.01, *** *p* < 0.001; → indicates significant time effect; *p*-value show significant group by time effect.

T Significant time effects were observed for maximal velocity (Vmax), total work (TW), and the related outcomes of PP, MP, and PD (*p* < 0.05). Results from separate analyses indicated that only within-group differences from baseline to post-intervention were significant in Vmax for the VBRT group (*p* < 0.001, effect size [ES] = 0.93) and in the related outcomes of PP (*p* < 0.01, ES = 0.5 to 0.68; see Figure 2A,B) and PD (*p* < 0.01, ES = 0.51 to 0.58; see Figure 2E) for the VBRT group. However, only the PBRT group showed significant improvements in MP (*p* < 0.01, ES = 0.38; see Figure 2C,D) and TW (*p* < 0.01, ES = 0.45; see Figure 2F). A significant “group × time” interaction effect was observed for MP and TW (*p* < 0.05; see Figure 2C,F). No significant group by time interaction effect was noted for the remaining anaerobic-related outcomes (see Table 3).

### 3.4. Jump and Sprint Performance 

**Figure 3:**
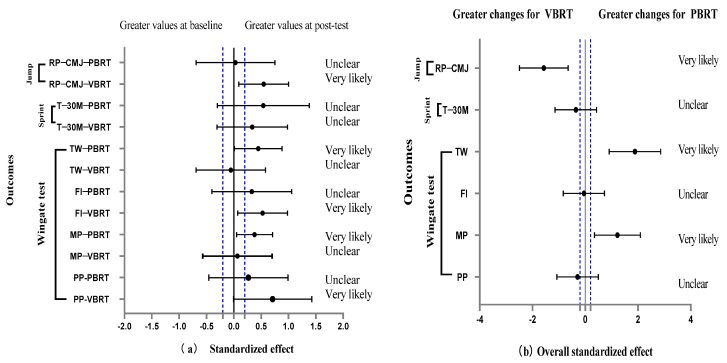
Standardized effect (90%CI) in all outcomes (**a**) between baseline and post−test and (**b**) between VBRT and PBRT groups. RP−CMJ = the relative power of countermovement jump, peak power(W)/body mass(kg); T−30 m = 30 m sprint running time; TW = total work; PP = peak power output; MP = mean power output; FI = fatigue index for the Wingate anaerobic test.

A significant time effect (*p* = 0.012) and group-by-time interaction effect (*p* = 0.018) were observed for RP-CMJ. The VBRT group showed a very likely improvement in RP-CMJ (7.0%; ES = 0.55) compared to baseline (Figure 3a). A comparison of between-group effects revealed that VBRT was more likely to be beneficial for RP-CMJ (Figure 3b). However, no group-by-time interaction and no significant improvement were found for T-30m (Table 3, Figure 3a). 

### 3.5. Training Effect

When comparing the power adaptation between the VBRT and PBRT (Figure 3), VBRT was very likely preferable for RP-CMJ (SMD = 0.55), whereas PBRT was very likely more beneficial for TW (SMD = 0.45) and MP (SMD = 0.38).

## 4. Discussion

The purpose of this study was to compare the effects of two load prescription methods, VBRT and PBRT, during a six-week progressive resistance training program on lower-limb power adaptation in female basketball players. The results showed that VBRT elicited greater improvements in RP-CMJ, Vmax, and PP compared to PBRT, which had more favorable effects on MP and TW. The findings support the notion that VBRT is effective in improving vertical jump performance, as evidenced by previous studies in various populations. The mechanisms behind these improvements may be attributed to the higher load and fewer repetitions implemented in the VBRT program, which reduced mechanical stress and fatigue while improving power output. 

The study evaluated the impact of two resistance training methods, individualized MCV-based and %1RM-based on CMJ height and T-30M. After a six-week intervention, the results showed that only the VBRT elicited improvements in RP-CMJ. This finding is supported by previous studies, including Orange et al. [11] which compared the effects of strength and jumping performance of rugby league players with seven-week VBRT and PBT, and found a likely improvement in CMJ height for both groups, although there were no significant differences between groups. Another study by Banyard et al. [9] found likely improvements in the fastest velocity of loaded CMJ (PV-CMJ) in 24 trained men with VBRT. The systematic review [18] also supports the use of velocity loss metrics as a monitoring tool in strength training, as it can improve CMJ height to varying degrees. 

The present study aimed to assess the effects of resistance training on lower-limb explosive power output using the relative power output of the CMJ as a direct variable. The findings suggest that VBRT is more effective than PBRT or different velocity loss interventions in improving vertical jump performance, including peak velocity and CMJ height. This has been observed in various populations, including players, fitness enthusiasts, and female athletes. The results provide valuable insights for sports that require high jumping ability, such as basketball and volleyball. The higher load and fewer repetitions mechanism of VBRT may explain its superior efficacy [19]. Additionally, routine strength training can lead to a decrease in velocity and power output with an increase in repetitions. High velocity-loss and high repetition resistance training can result in a significant reduction in type IIX fibers, which may negatively impact strength development and prolong recovery [20]. Velocity-based prescription and monitoring velocity loss can mitigate these issues by ensuring higher quality completion of each repetition and reducing unnecessary mechanical stress and fatigue [18]. The present study highlights the importance of heavy intensity and slightly fewer repetitions in enhancing explosive power.

The results regarding the effects on sprint ability in both groups are inconclusive due to the lack of plyometrics and linear sprint training in the six-week intervention. Previous studies have shown mixed results. Banyard et al. study on adult trained males (n = 24) reported that VBRT improved 10 m and 20 m sprint performance (10 m: ES = 1.24; 20 m: ES = 1.27). However, a larger study on youth football players over 12 weeks of maximum velocity (VG) and maximal strength (RMG) training found that only VG improved 30 m sprint performance (*p* < 0.001; ES = −1.26) [21]. Further research is required to determine whether VBRT can improve sprint performance. 

Currently, most studies on WAnT have focused on the acute effects of non-strength training interventions. The limited and complex nature of laboratory-based studies has resulted in a scarcity of research examining the impact of resistance training on anaerobic performance. In a study by Rønnestad et al. [22], 16 elite cyclists were subjected to 25 weeks of combined endurance and percentage-based half-squat training (E + S) or endurance training only (E). Results showed a moderate effect of E + S on relative peak power (PP) in the WAnT (ES = 0.76). Pallares et al. [23] also demonstrated the effectiveness of VBRT through improved parallel-squat PP and MP) (ES = 0.15–0.56). While these studies suggest that VBRT or PBRT may have a positive effect on anaerobic performance, the underlying mechanism remains unclear. Moreover, there is a lack of direct comparative research in this field.

The anaerobic metabolic capacity is influenced by several factors including age, gender, and muscle mass. PP during the WAnT reflects the energy-generating capacity of the ATP-CP system and is dependent on muscle mass and maximum leg strength [24], particularly the amount of FFM. MP during the test reflects the energy generated from glycolysis and represents muscle endurance [25]. Previous research has shown that high velocity-loss RT can increase muscle cross-sectional area (CSA) and muscle volume, particularly in the vastus lateralis and intermedius muscles, with the use of higher loads and fewer repetitions [26]. However, the current study did not observe significant changes in FFM and muscle mass, which may be due to differences in the number of repetitions and overall training volume compared to previous studies. Thus, it is inappropriate to draw conclusions on the relationship between neuromuscular adaptations and muscle mass or hypertrophy based on the results of this study.

The present study aimed to investigate the relationship between muscle fiber type and anaerobic capacity, as manifested in the percentage of fast-muscle fibers. Our findings showed a positive correlation between the high percentage of fast-muscle fibers and high anaerobic power output during contraction, as well as maximum knee extension force, contraction force, and velocity. The dominance of fast muscle fibers in maximum instantaneous power output and short-duration anaerobic power was also observed. In exercises that heavily rely on anaerobic metabolic energy, faster muscle fibers or larger cross-sectional area can sustain maximal power output for a relatively longer period. Lahti et al. [27] demonstrated that high-intensity training, such as the VBRT employed in this study, can induce a shift in fiber type towards type IIa fast muscle fibers and enhance explosive power in athletes. Therefore, athletes can increase the proportion of fast fibers through training and thereby shift the force-velocity curve toward the right and upward. In the overview of the literature and theory, the higher intensity and lower volume prescription observed in this study during VBRT group may explain the observed increase in PP and V-max, as well as RP and peak velocity of the CMJ were also considered. It seemed possible that these results were also due to the feedback during RT that inspired the athletes to accomplish concentric velocity in VBRT [28], which contributed to heavier lifting weights, as well as faster and fewer repetitions, thus possibly inducing a shift in type IIa fast muscle fibers or a positive shift in the force-velocity curve. Myofibrillar subclasses can be converted rather quickly into a more active type II subclass through RT programs, and high-intensity exercises appear to facilitate the conversion of myofibrillar subclasses from type IIx to type IIa. Higher VL allowed for greater volume, thus maximizing muscle hypertrophy, while the lower VL triggered positive neuromuscular-specific adaptations [26]. Our results also showed that VBRT induced greater adaptation of faster and less fatigue-resistant muscle fibers, whereas the PBRT resulted in higher muscle endurance and time to exhaustion but had less impact on power output. Further studies are needed to examine the differences in changes in force-velocity relationship between these two strength methods. This research has practical implications for athletic performance and neuromuscular adaptations, as it provides novel insights into the differences between VBRT and PBRT from an anaerobic power capacity perspective.

The finding suggests that the two strength training methods, VBRT and PBRT, induce distinct neuromuscular adaptations. Both high power anaerobic endurance and instantaneous explosive power are key physical fitness factors crucial for basketball performance. By monitoring real-time muscle contraction velocity during strength training, a method can be prescribed that utilizes higher intensity loading weights, promotes specific adaptations, and reduces fatigue. This study sheds light on the underlying mechanisms of action of both VBRT and PBRT, reducing the potential for subjective bias from physical fitness experts and athletes. As a result, it provides a basis for choosing the appropriate strength training method for specific training goals, rather than dismissing previous training approaches.

This study found that VBRT was effective in improving PP, V-max, and RP-CMJ in basketball players. These results provide new insights into the differential impact of VBRT and PBRT and could aid in the selection of strength training programs for sports that require high jumping ability. The use of velocity-based prescription and monitoring in resistance training programs was emphasized by these findings, highlighting their practical significance in improving athletic performance in sports such as basketball and volleyball.

The limitations of this study include the inability to determine changes in muscle fiber type or percentage distributions and a small sample size. These limitations highlight the need for future research with a larger sample size to confirm the transformation of fiber types. Despite these limitations, the study should be acknowledged for its contribution to the field of sports science by exploring the influence of VBRT on female basketball players, which is an understudied area [29]. Further research is needed to expand upon the results and address the limitations of the study.

## 5. Conclusions

The results of this study demonstrate that after six weeks of back squat training with different load prescription protocols, VBRT and PBRT are different as regards improving physical parameters related to basketball performance. Specifically, VBRT was effective in improving PP, V-max, and RP-CMJ, while PBRT was more beneficial in improving MP and TW. These findings suggest that PBRT might target high power velocity endurance, whereas VBRT might primarily induce greater instantaneous mechanical power for explosive adaptations.

## Figures and Tables

**Table 1:** Participants characteristics.

Characteristic	VBRT	PBRT	*p*-Value	SMD
Age (y) *	22.6 ± 1.6	22.0 ± 2.0	0.49	0.32
Training years (y) *	7.8 ± 3.4	8.2 ± 2.6	0.85	0.1
Height (cm) *	169.8 ± 6.0	169.4 ± 8.1	0.9	0.06
Body mass (kg) *	60.0 ± 4.8	61.0 ± 7.7	0.92	0.04
Fat mass (kg) *	21.8 ± 6.1	21.5 ± 4.6	0.92	0.05
Muscle mass (kg) *	43.1 ± 3.1	45.1 ± 4.6	0.3	0.49
BMI *	21.7 ± 1.9	21.4 ± 2.1	0.78	0.13
Fat Free Mass (FFM) *	45.9 ± 3.3	46.6 ± 4.1	0.7	0.04
FMS test ~	16 (13–16)	15 (13–16)	0.39	0.39
The deep squat ~	2 (2–3)	2 (2–3)	1	0
Back squat 1-RM (kg) *	81.0 ± 12.4	85.9 ± 6.9	0.33	−0.45
R-SQ 1RM *	1.3 ± 0.2	1.4 ± 0.2	0.34	−0.45
Total work (J) *	12,492.2 ± 1294.4	12,262.3 ± 1709.1	0.75	0.15

Data are median (range) or mean ± SD. *p*-values for * *t*-test and ~ Mann–Whitney U-test are applied to test for differences between VBRT and PBRT groups.

**Table 2:** Results of lower limb muscle mass and morphological development.

Outcome	VBRT (n = 10)		SMD (95%CI)	PBRT (n = 8)		SMD(95%CI)	RM-ANOVA	
	Baseline	Post-Intervention		Baseline	Post-Intervention		Time	Group × Time
BM (kg)	60.0 ± 4.2	59.6 ± 5.0	−0.08 (−0.22, 0.03)	61.0 ± 7.7	61.2 ± 7.8	−0.02 (−0.07, 0.11)	0.636	0.305
FFM (kg)	45.9 ± 3.3	46.5 ± 4.0	0.15 (−0.22, 0.56)	46.6 ± 4.1	46.5 ± 4.2	−0.03 (−0.17, 0.11)	0.557	0.391
BMI (kg/m^2^)	21.7 ± 1.9	21.5 ± 1.9	−0.1 (−0.24, 0.02)	21.4 ± 2.1	21.4 ± 2.0	−0.01 (−0.12, 0.09)	0.189	0.308
MM (kg)	43.1 ± 3.1	43.1 ± 3.6	0 (−0.20, 0.20)	45.1 ± 4.6	44.8 ± 4.7	−0.05 (−0.14, 0.04)	0.609	0.609
HC (cm)	93.3 ± 2.5	93.1 ± 3.0	−0.08 (−0.24, 0.06)	92.8 ± 4.1	92.6 ± 4.1	−0.04 (−0.14, 0.03)	0.174	0.887
LLC (cm)	50.2 ± 1.3	50.0 ± 1.5	−0.17 (−0.48, 0.1)	50.4 ± 3.2	50.4 ± 3.1	−0.02 (−0.11, 0.07)	0.256	0.443
RLC (cm)	50.3 ± 1.4	50 ± 1.5	−0.2(−0.55, 0.09)	50.7 ± 3.3	50.5 ± 3.2	−0.04 (−0.14, 0.04)	0.116	0.571

Abbreviations: BM = body mass; FFM = Fat Free Mass; BMI = body mass index, BMI = mass (kg)/height^2^ (m); MM = muscle mass; HC: hip circumference; LLC, RLC: left leg circumference, right leg circumference.

**Table 3:** Results of mechanisms of lower limb muscle anaerobic performance adaptation between VBRT and PBRT.

Outcome	VBRT		SMD (95%CI)	Inference	PBRT		SMD (95%CI)	Inference	RM-ANOVA	
	Baseline	Post-Intervention			Baseline	Post-Intervention			Time	Group × Time
30-m sprint (s)	4.78 ± 0.2	4.71 ± 0.2	−0.46 (−0.87, −0.16)	71/28/1	4.8 ± 0.1	4.71 ± 0.2	−0.2 (−1.35, 0.87)	55/20/25	0.053	0.197
				Unclear				Unclear		
RP-CMJ (W/kg)	45.7 ± 5.2	48.7 ± 5.3 **	0.55 (0.25, 0.99)	98/2/0	47.3 ± 3.5	47.4 ± 2.8	0.03 (−0.43, 0.51)	22/62/16	0.012	0.018
				Very Likely				Unclear		
T-PP (ms)	2976.5 ± 853.7	2932.5 ± 2817.7	−0.03 (−0.77, 0.7)	54/35/11	2416.3 ± 995.4	2478.8.2 ± 1367	0.05 (−0.67, 0.55)	62/25/14	0.639	0.568
				Unclear				Unclear		
V-max (m/s)	147.8 ± 13.1	160.5 ± 13.0 ***	0.94 (0.54, 1.37)	99.9/0.1/0	145 ± 17.8	154.2 ± 14.4	0.54 (−0.06, 1.31)	87/11/2	0.001	0.501
				Almost				Possibly		
TW (J)	12,492 ± 1294	12,420 ± 1318	0.05 (−0.34, 0.22)	4/81/15	12,262 ± 1709	12,998 ± 1388 **	0.45 (0.25, 0.80)	96.5/3.4/0.0	0.018	0.006
				Unclear				Very Likely		
PP [W]	572.7 ± 43.9	607.4 ± 54.0 **	0.68 (0.31, 1.21)	99/1/0	592.1 ± 87.4	615.3 ± 76.5	0.27 (−0.11, 0.74)	63.3/34.7/2.0	0.001	0.524
				Very Likely				Unclear		
RPP [W/kg]	9.2 ± 1.0	9.8 ± 1.3 *	0.5 (0.13, 1.31)	96/4/0	9.7 ± 0.8	10.2 ± 0.8	0.65 (−0.03, 1.58)	83.7/13.2/3.1	0.006	0.672
				Very Likely				Possibly		
MP [W]	432.8 ± 48.3	436.3 ± 51.4	0.07 (−0.16, 0.31)	15/83/2	427.8 ± 63.1	451.8 ± 55.5 **	0.38 (0.23, 0.66)	97.4/2.6/0.0	0.002	0.016
				Unclear				Very Likely		
RMP [W/kg]	7.1 ± 0.7	7.2 ± 0.9	0.16 (−0.09, 0.46)	49/49/2	7.0 ± 0.6	7.4 ± 0.5 ***	0.7 (0.41, 1.22)	99.7/0.3/0.0	0.002	0.079
				Unclear				Almost		
PD [W]	300.8 ± 47.0	331.0 ± 62.0 **	0.53 (0.26, 0.93)	98/2/0	351.6 ± 87.9	379.4 ± 69.6	0.33 (−0.28, 1.07)	65.1/29.4/5.5	0.022	0.916
				Very Likely				Unclear		
PD [W/kg]	4.9 ± 0.9	5.5 ± 1.1 **	0.5 (0.26, 0.88)	99/1/0	5.6 ± 0.9	6.1 ± 1.0	0.59 (−0.26, 1.49)	80.5/14.1/5.4	0.008	0.981
				Very Likely				Possibly		
PD [W/s]	10 ± 1.6	11 ± 2.1 **	0.53 (0.26, 0.93)	98/2/0	11.7 ± 2.9	12.6 ± 2.3	0.33 (−0.28, 1.07)	73.9/17.7/8.5	0.022	0.916
				Very Likely				Possibly		
PD [%]	53.3 ± 5.3	55.7 ± 5.6	0.41 (−0.11, 1.05)	80/18/2	56.8 ± 7.2	60.5 ± 6.4	0.51 (−0.47, 1.7)	73.9/17.7/8.5	0.083	0.679
				Possibly				Possibly		

Data are mean ± SD. * indicates significances difference = * *p* < 0.05, ** *p* < 0.01, *** *p* < 0.001. Inferences = magnitude-based inference; RM-ANOVA = repeated-measures analysis of variance; RP-CMJ = the relative power of countermovement jump, peak power (W)/body mass (kg); T-PP = time to peak power; TW = Total work; PP, RPP = absolute peak power and relative peak power output; MP = absolute mean power and relative mean power output; PD = power drop for the Wingate anaerobic power test; VBRT = velocity-based resistance training; PBRT= (%1RM) percentage-based resistance training. CI = confidence interval; SMD = standardized mean difference; Inference = magnitude based.

## Data Availability

The original contributions presented in the study can be directed to the corresponding authors. Written informed consent has been obtained from the patients to publish this paper.
